# Treating insomnia in people who are incarcerated: a feasibility study of a multicomponent treatment pathway

**DOI:** 10.1093/sleepadvances/zpae003

**Published:** 2024-01-20

**Authors:** Lindsay H Dewa, Bethan Thibaut, Natalie Pattison, Sean James Campbell, Thomas Woodcock, Paul Aylin, Stephanie Archer

**Affiliations:** Imperial College London, School of Public Health, London, UK; Imperial College London, School of Public Health, London, UK; Care UK, Health in Justice, London, UK; User Voice, London, UK; Imperial College London, School of Public Health, London, UK; Imperial College London, School of Public Health, London, UK; University of Cambridge, Department of Public Health and Primary Care, UK

**Keywords:** prison, incarceration, sleep, insomnia, treatment pathway, peer support, cognitive behavioral therapy, stepped-care, non-pharmacological

## Abstract

Around 60% of people who are incarcerated have insomnia; 6–10 times more prevalent than the general population. Yet, there is no standardized, evidence-based approach to insomnia treatment in prison. We assessed the feasibility of a treatment pathway for insomnia in a high-secure prison to inform a future randomized controlled trial (RCT) and initial efficacy data for sleep and mental health outcomes. We used a within-participants pre-post design. The stepped-care pathway included: self-management with peer support, environmental aids, and cognitive behavioral therapy for insomnia (CBTi). Assessment measures for insomnia, well-being, mood, anxiety, suicidality, overall health, sleepiness, fatigue, and cognitive functioning were administered at baseline and pathway exit. Feasibility criteria included eligibility to participate, CBTi uptake, and assessment completion. Forty-two adult males who are incarcerated were approached of which 95.2% were eligible. Of those deemed eligible, most participated (36/40, 90.0%). Most who completed baseline completed post-assessments (28/36, 77.8%) and of these, most showed improvements in their subjective sleep (27/28, 96.4%). Large reductions were found from pre- to posttreatment in insomnia severity (*d* = −1.81, 95% CI: 8.3 to 12.9) and 57.0% reported no clinically significant insomnia symptoms at post-assessment. There was no overall change in actigraphy-measured sleep. Large treatment benefits were found for depression, anxiety, well-being, and cognitive functioning, with a medium benefit on suicidal ideation. The treatment pathway for insomnia in prison was feasible and may be an effective treatment for insomnia in people who are incarcerated, with additional promising benefits for mental health. A pragmatic RCT across different prison populations is warranted. This paper is part of the *Sleep and Circadian Health in the Justice System* Collection.

**Statement of Significance**

Our study is the first, worldwide, to examine feasibility and initial efficacy of using a non-pharmacological intervention for chronic insomnia in a challenging prison environment. The treatment pathway is feasible and may improve subjective sleep and reduce depression and anxiety symptoms. Suicidal ideation may also be reduced after the intervention. This is encouraging because the pathway could help overcome medication trading, misuse, and barriers to on-wing administration and lead to further evaluation of the potential effects of improved sleep on mental health, well-being, and suicidality. Our reported meaningful patient and public involvement (PPI) has implications for clinical practice; working with patients as equal research partners is essential for quality, efficiency, and research impact, but may also improve sleep interventions internationally.

## Introduction

Just over 60% of people who are incarcerated in the UK have insomnia, which is 6–10 times more prevalent than in the general population [1, 2]. This is concerning because insomnia and general sleep disturbance are significantly associated with cardiovascular disease [3], chronic pain [3], poor quality of life [4] and psychiatric disorders [5], and mortality [6] which are all disproportionately likely in people who are incarcerated. Moreover, there is a well-recognized relationship between sleep disturbance and suicidal ideation, behavior, and deaths due to suicide [7–9]. It is therefore plausible that insomnia may increase the vulnerability to suicide in prison populations around the globe [10], and that treating insomnia may decrease this risk.

Having a good night’s sleep in prison can be difficult. Fear of violence from others in prison at night can trigger presleep hyperarousal making people more likely to have poor sleep. In contrast, sleep disturbance is also associated with irritability and aggressive behavior [11, 12] which are both prevalent in incarcerated individuals [13]. If sleep disturbance is causally related to aggression [11, 14], this has clear implications for the safety of people who are incarcerated and staff, and in the longer term, untreated insomnia could theoretically increase the likelihood of re-offending.

Limited natural light and physical activity reduce circadian zeitgebers, and excessive noise and nighttime observations directly disrupt sleep [15, 16]. People who are incarcerated are often locked up for up to 23 hours a day and subsequently nap in the daytime [17] which reduces homeostatic sleep pressure at night. Prison cells can become associated with wakefulness, not sleep, which can contribute to stimulated arousal at bedtime [17]. Prison and resource constraints also reduce opportunities for typical relaxation routines (e.g. watching television or taking a bath). The high insomnia prevalence and severe health- and safety-related consequences suggest that optimizing sleep treatments, and/or addressing factors that lead to sleep problems, could be important targets in prison.

To treat mental health conditions and/or help patients achieve better sleep, hypnotic and anxiolytic medication (e.g. benzodiazepines) are often prescribed which cause sedation. These prescriptions are 10 times higher in prison compared to the general population [18, 19]. While medication may be prescribed to help patients sleep, it is often administered at inappropriate times (e.g. in the afternoon), because healthcare professionals do not have access to patients before bedtime due to the lockdown on prison wings [20]. Inappropriate administration causes sleep-onset at the wrong time of the day which can further disrupt the natural sleep–wake cycle and underlying circadian rhythmicity [21]. Medication also poses both a health and security risk that can result in medication misuse, trading, and selling [22]. As such, alternative non-pharmacological approaches may be more appropriate to treat insomnia in prison.

International treatment guidelines state that cognitive behavioral therapy for insomnia (CBTi) (i.e. a multicomponent psychological intervention) is the first line recommended intervention for persistent insomnia [23, 24]. Meta-analyses have confirmed the efficacy of CBTi with the inclusion of self-management modalities [25] and adaptations for populations, methods, and settings [26]. However, prisons currently offer an unstandardized approach to insomnia treatment which is not consistent with current treatment guidelines; for example, a national survey of UK prisons revealed just one prison offered CBTi [20]. Instead, prisons mainly offer sleep hygiene education (i.e. a list of behaviors to follow to promote good sleep) and medication [20].

While CBTi has been shown to be effective in other settings [26] few trials into the effectiveness of CBTi have been conducted in a prison setting [15, 27]. To the best of our knowledge, there has been just one uncontrolled study, which has tested a psychological sleep intervention in prison where a single session (60–70 minutes) of CBTi resulted in improved sleep (*d*_z_ 2.35) [28]. However, this study had a short follow-up period, recruited people who were incarcerated with acute insomnia and specifically excluded chronic insomnia, despite this being the more common presentation. Our work builds on this study and our initial pathway for insomnia in prison [29], by developing a non-pharmacological intervention for chronic insomnia, informed by stakeholders, and tested over time. The study primary aim was to test the feasibility of a CBTi-based treatment pathway in a high-secure prison setting in England to inform a full randomized controlled trial (RCT). The secondary aim was to collect initial efficacy data on sleep, mental well-being, and other health-related outcomes in people who are incarcerated.

## Materials and Methods

### Study design and setting

The feasibility study used a within-participants pre-post design. The study was set in an Increasing Access to Psychological Therapies (IAPT) service in one UK high secure^1^ male prison where people who are incarcerated are serving 5 or more years, primarily for sexual and violent offences. People who are incarcerated sleep in individual cells and are monitored throughout the night by prison officers.

### CBTi intervention design and specification

**Figure 1.**
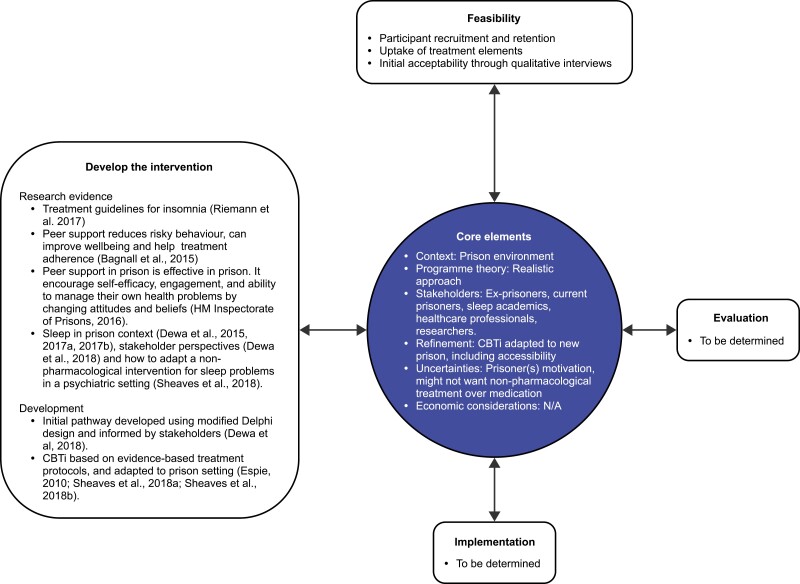
Framework showing main phases and core elements of the treatment pathway adapted from the MRC Framework for development and evaluation of complex interventions [30]

The treatment pathway for insomnia in prison is a multicomponent intervention which was informed by the Medical Research Council framework for development and evaluation of complex interventions [30]. This includes development (published elsewhere [29]); feasibility, evaluation, and implementation (Figure 1). The initial pathway developed using a modified Delphi design [29] was further modified (see Patient and Public Involvement (PPI) section), and resulted in four main stages: self-management with peer support, environmental aids, selective hypnotic prescribing and CBTi, adapted to suit the prison setting (Table 1). However, some patients could have self-management with peer support *and* environmental aids, and some could go through all stages, including CBTi if their sleep problem persisted.

**Table 1:** Key Adaptions of a Non-pharmacological Insomnia Treatment for the Prison Setting

Non-pharmacological component	Problem	Adaption
Sleep hygiene	People who are incarcerated have nighttime observations meaning prison cell slats open suddenly and the light is turned on or a torch is used. This can occur at various times during the night.	Eye masks and earplugs were given to those affected by these prison environmental issues.
Stimulus control	It is common for people who are incarcerated to be in their cells for 23 hours a day. As such people who are incarcerated nap during the day, lie on their beds and have limited access to natural light. This negatively impacts on their sleep–wake cycle. The association between bed and sleep is weakened.	The association between bed and sleep was encouraged by changing the cell set-up to reflect time of day (e.g. arrange cell to be a living room in day and bedroom at night).
Sleep restriction	Typically, sleep restriction reduces the opportunity for sleep such that it is in line with the current total sleep time. However, this was a vulnerable population who may be more sensitive to the effects of sleep loss.	The current protocol reduced the opportunity for sleep to the people who are incarcerated’ goal total sleep time.
Ongoing sleep support	Clinicians are unable to access the prison wings at night and usually meet people who are incarcerated at scheduled appointments.	Participants had access to sleep peer support on the wings, available when people who are incarcerated are unlocked from their cells. Two sleep peer workers were trained in basic sleep management in each wing. They met with all participants at entry to the pathway and agreed on a plan going forward.

The treatment pathway had several complex steps. First, following a positive screen for insomnia, all people who were incarcerated were given a coproduced self-management sleep booklet and access to peer support. The booklet outlined (1) basic sleep hygiene adapted to the prison setting (e.g. physical exercise and relaxation techniques in cell), (2) how to achieve a good sleep environment in the cell (e.g. arranging cell according to daytime and bedtime), and (3) how to manage worry to promote better sleep in prison. Two security-cleared people who were incarcerated were trained in each of the four wings in basic sleep management techniques and given supplementary training booklets, to offer peer support on wings. The peer support workers gave out the sleep booklets and went through it with the participants; they generally helped encourage engagement in a new sleep routine on the wings, when clinicians were unavailable. The therapist (NP) provided monthly support to the peer support workers if needed.

Second, environmental aids (earplugs and/or eye mask) were then offered to people who were incarcerated if their sleep was disturbed by light and/or noise. Third, hypnotic medication was offered if a person who was incarcerated presented with acute (rather than persistent) insomnia, in line with UK National Institute for Health and Care Excellence (NICE) guidelines [31]. This was indicated by a score below clinical cutoff (scored 3 or 4) on question 8 (how long have you had a problem with your sleep?) and accompanied by an acute stressor (e.g. experience of bereavement, unexpected or long sentence). In practice, no one presented with acute insomnia and hence this was not offered.

Lastly, if people who were incarcerated still presented with insomnia after these lower-intensity interventions, they were offered CBTi as part of their long-term insomnia management. CBTi sessions took place with an IAPT therapist (NP) with local service supervision and occasional specialist supervision from a clinical psychologist. CBTi therapy was designed to be delivered weekly and over 6–8 sessions, but some flexibility was required to ensure session delivery considering prison schedule changes, patient availability, and prison lockdowns which meant patients could not be seen. Sessions were rescheduled at the earliest opportunity. Workbook-style manuals were shared between patient and therapist for delivery of techniques. The full intervention components included:

- Assessment and goal setting.
- Psychoeducation about sleep architecture, homeostatic sleep pressure, and circadian rhythmicity.
- Stimulus control (i.e. promoting associative learning between bed and sleep and breaking the association between lying in bed and wakefulness).
- Sleep hygiene (including minimizing nighttime disruptions in the prison by promoting the use of ear masks, ear plugs and making sure the prison cell was dark).
- Adapted sleep restriction (i.e. setting a consistent sleep window in line with the goal sleep duration and chronotype of the patient).
- Targeting hyperarousal via relaxation and strategies to combat nighttime worry.
- Managing fatigue (including promoting more exposure to daylight, keeping active, and maintaining a healthy diet).
- Relapse management.

The sleep condition indicator (SCI) was used as a clinical tool to help inform pathway progression [32]. For example, if the SCI remained low or was worse after self-management and peer support then it was likely that they went on to more intensive CBTi. Patients could also leave the pathway at different stages if their sleep had improved to the satisfaction of both the patient and therapist.

### Participants

Participants were eligible if they satisfied the following criteria: aged ≥18 years; had a diagnosis of insomnia disorder (Diagnostic and Statistical Manual of Mental Disorders version 5; DSM-V) or positive screen (a score of ≤16) on the SCI [32]; resided in the same prison for at least 6 months and had an adequate understanding of the English language for completing assessments and therapy. Exclusion criteria were the presence of risk markers indicating that face-to-face administered questionnaires could not be conducted alone; too physically or mentally unwell to participate; or unable to provide informed consent. No monetary incentive was given to participate in the study. All participants gave informed written consent.

### Procedure

Recruitment took place over 2 months (April 25th to June 27th, 2018). Participants who reported a sleep complaint to peer-support workers or prison healthcare staff were referred to a CBTi therapist (NP) who assessed for eligibility criteria and willingness to take part in the study. An independent researcher (BT) obtained consent and administered face-to-face assessments (Table 2) with each participant in a private room in the prison at baseline (0 weeks) and at exit from the pathway. Post-assessments were completed as soon as practically possible upon patients exiting the pathway and were initially planned to be conducted at 2 weeks for self-management with peer support, 3 weeks for those who additionally used environmental aids, and 12 weeks for those who went on to receive CBTi. Objective assessment of insomnia (i.e. actigraphy watch) and corresponding sleep diaries were measured in the first 10 eligible participants for 7 days at baseline and post-assessment. Following the baseline assessment all participants entered the pathway and started treatment. After the pathway exit, a purposeful sample of participants and peer-support workers were interviewed about their experience using the pathway. Semi-structured interviews were audio recorded and transcribed verbatim, and will be reported on in a separate paper.

**Table 2.** Subjective Self-report Assessments

Concept	Scale	Scoring and measuring description	Justification
Insomnia	Sleep condition indicator (SCI) [32]	Eight items; were rated on a 4 to 0 scale (higher scores indicated better sleep). Items are totaled and a score ≤16 is indicative of possible insomnia disorder (range 0–32).	Good internal consistency (*α* = 0.89), good concurrent validity, and sensitivity to change following CBTi.
Subjective sleep quality	Pittsburgh sleep quality index (PSQI) [33]	Nineteen items; rated on a scale from 0 to 3. Higher total score reflects poorer sleep (range 0–57).	Good internal consistency (*α* = 0.89) and has previously been used in a prison setting.
Psychological well-being	Warwick-edinburgh mental well-being scale (WEMWBS) [34]	Fourteen items; rated on a scale from 1 (none of the time) to 5 (all of the time). Higher the total score the higher the well-being (range 14–70).	Excellent internal consistency (0.95), valid and responsive to change, including in a prison population [35]
Mood	Patient health questionnaire (PHQ-9) [36]	Nine items; were rated on a 0 (not at all) to 3 (everyday). Higher scores indicate more severe depression (range 0–27). cutoff score for possible depression is ≥10.	Excellent internal reliability (*α* = 0.89), sensitivity, and specificity (88%; scores ≥10) and used in clinical practice across all prisons in England and Wales.
Anxiety	Generalized anxiety disorder assessment (GAD-7) [37]	Seven items; rated on a 0 to 3 scale (not at all to nearly every day, respectively). Higher the score the more severe the anxiety. cutoff score for moderate anxiety is ≥10.	Excellent internal consistency (*α* = 0.92), sensitivity (89%), and specificity (82%) and used in clinical practice across all prisons in England and Wales.
Suicidal ideation	Beck scale for suicide ideation (BSS) [38]	Twenty-one items; rated on a 0 to 2 scale. Higher scores reflect greater levels of suicidal ideation (range 9–19).	Good to excellent internal consistency (*α* = 0.84 to *α* = 0.93). A commonly used measure of suicidal ideation.
General Health	Patient-reported Outcomes Measurement Information System (PROMIS-10) [39]	Ten-item; mostly rated on 0-5 scale, one question has 0–11 scale. Higher scores reflect better general health.	Good internal consistency (*α* = 0.81), easy to use, and short.
Sleepiness	Epworth sleepiness scale (ESS) [40]	Eight-item; rated on a 0 to 3 scale. The higher the score the higher chance of sleepiness (range 0–24). After review and discussion with a person with prison experience, item 8 was omitted because it reflected something was unlikely to happen in a prison setting (e.g. "In a car, while stopped for a few minutes in the traffic").	Excellent internal consistency (*α* = 0.91), and a commonly used assessment for sleepiness.
Fatigue	Flinders fatigue scale (FSS) [41]	Seven items; were rated on a 0 to 3 scale. Higher scores reflect more daytime fatigue (range 0–21).	High internal consistency (*α* = 0.91).
Cognitive functioning	Cognitive Failures Questionnaire (CFS) [42]	Twenty-five items; rated on a scale of 0–4. Higher scores indicate a higher chance of cognitive lapse (range 0–72). After review and discussion with a person with prison experience, items 2, 3, 4, 8, 12, and 13 were omitted because they reflected things that were unlikely to occur in a prison setting (e.g. “Do you fail to notice signposts on the road?”).	Good test–retest reliability (*r *= 0.71).

### Objective sleep measure

Actigraphy is a reliable objective measure for assessing the sleep-activity pattern [43]. The GENEActiv Original (Activinsights Ltd, United Kingdom) is a water-resistant and lightweight accelerometer, validated for sleep research [44, 45], that has a built-in frequency of 32.768 kHz and +/− 20 ppm (+/− 1.7s/day) on a microelectromechanical systems (MEMS) sensor (+/− 8 g). Participants were asked to wear GENEActiv for 24 hours for 7 days pre and post-assessment. Manufacturer scoring algorithms were used. Activity was captured using 60-second epochs to detect rest and sleep periods. Automated accelerometer sleep windows were used, in line with other studies, due to the amount of missing data for the post-assessment sleep diaries. Selected participants were instructed to wear the accelerometer for 7 days on their non-dominant hand with the watch face facing upwards, a week before and after the pathway. It was used to monitor movement, rest, and light exposure (+/− 10% @ 1000 Lux calibration).

### Study feasibility outcomes

The primary aim of our study was to assess the feasibility of a treatment pathway in a high-secure prison setting to inform a future RCT. Specifically, we sought to assess the recruitment and retention of participants, and the uptake of treatment elements.

### Patient and public involvement

We worked together with people with professional or lived experience of insomnia and prison-based interventions (previous or current experience) across the following research stages: identifying and prioritization, commissioning/funding, design, management, data collection, and dissemination. User Voice, a user-led organization where people with lived experience of prison are staff or researchers, led and delivered all patient and public involvement (PPI), attended all steering group meetings, and shared power and decisions with the team throughout. On September 7th, 2017, the chief investigator (LD) held a full day session with User Voice to develop self-management sleep booklets and peer support training booklets. The opportunity to further improve the sleep booklets was advertised to men with current lived prison experience, residents in the high-secure prison, in October 2017. Five signed up and became a prison advisory group. The prison advisory group attended three face-to-face 2-hour consultations in the high-secure prison, along with the chief investigator (LD), and two healthcare practitioners (one from the Drug and Alcohol Recovery Team and one from the IAPT team) to further inform the treatment pathway and sleep booklets. Amendments were made after every meeting following suggestions from the group (e.g. language was put into plain English). User Voice are also coauthors of this paper. All learning, challenges, and possible solutions were recorded and will be published separately.

### Serious adverse events

Serious adverse events (SAE) were predetermined and included deaths by suicide, attempted suicide, self-harm, serious interpersonal violence, and admission to hospital outside prison. If the CBTi therapist (NP) or research assistant (BT) became aware of a SAE during the study, the protocol was to report to the chief investigator (LD) who would have notified the sponsor. Clinical and prison databases were reviewed by NP or BT at the end of the study for SAEs that may not have been detected by staff. In cases of concern for immediate danger of self-injury NP and BT would have opened an Assessment, Care, Custody, and Teamwork (ACCT) form.^2^

### Statistical analysis

Descriptive statistics were reported for demographic, clinical, and forensic factors (numbers and percentages) in all participants who completed the baseline assessment. All participants who completed both baseline and post-assessments were included in subsequent analyses. Paired *t*-tests were then employed comparing the primary and secondary subjective health-related measure scores before and after the intervention. Within-group standardized effect sizes using Cohen’s *d* were also calculated for SCI, PSQI, WEMWBS, PHQ-9, GAD-7, PROMIS-10, ESS, BSS, FSS, and CFF. Given the objectives of this feasibility study, the analysis plan did not include reporting of *p* values, instead effect sizes and 95% confidence intervals are reported. Effect size definitions were small (*d = *0.20), moderate (*d* = 0.50), and large (*d = *0.80).

Actigraph data were collected and stored in epochs. We collected 7 days of actigraph data at baseline and pathway exit. Raw data were converted from binary to CSV files and processed in Excel. Sleep parameters captured included: total sleep time, sleep-onset latency, wakefulness after sleep-onset (awakening duration during rest period), and sleep efficiency.

### Research governance and ethics

The study was reviewed and approved by NHS Research Ethics Committee for Wales (REC ref: 17/WS/0238) and Her Majesty’s Prison Probation Service (HMPPS) (NRC 2017-319).

## Results

### Overview of baseline participants

**Figure 2.**
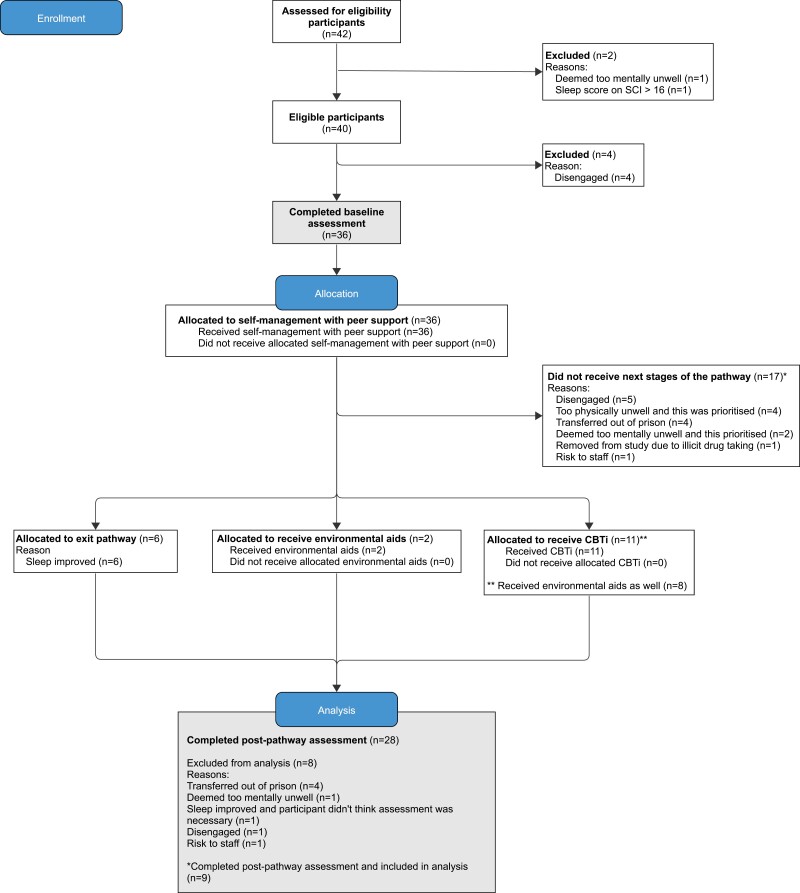
Flowchart of participants adapted from Consolidated Standards of Reporting Trials (CONSORT) diagram [46]

Forty-two participants were approached, and the majority were deemed eligible (40/42; 95.2%). Most were recruited and completed the baseline assessment (36/40; 90.0%) (Figure 2). Of these, the mean age was 42.1 (±12.6; range 23–65 years), they were mostly White-British (91.7%), single (75.0%), and incarcerated due to a sexual offense (52.8%) (Table 3). They had been in prison for an average of 4.8 years. Two-thirds had trouble sleeping before coming into prison (24/36; 66.7%); all of these had experienced trouble sleeping in their home environment (24/24; 100%), and just over a third (7/20; 35.0%) had experienced difficulties with sleeping in another prison. The majority had persistent insomnia (35/36, 97.2%), were taking at least one medication for physical or mental health difficulties (29/36, 80.6%), and screened positive for depression and anxiety (32/35^3^; 91.4% and 24/36; 66.7%, respectively). One CBTi participant had a significant change in medication (cessation of codeine medication). This withdrawal can exacerbate sleep problems. One participant was prescribed hypnotic medication for three nights by a GP without consultation with research team. Data from each of these participants was included in the analysis. Care offered to study participants continued as usual: some participants received recovery service 1-1 work on emotional management and drug use. Others had treatment for physical health conditions including physiotherapy, diabetes, and cardiac treatment.

**Table 3.** Baseline Participant Characteristics (*N* = 36)

Characteristic	*n* (%)
Age^a^	42.1 (12.6)
*Ethnicity*	
White-British	33 (91.7%)
Mixed	2 (5.6%)
Chinese or other	1 (2.8%)
*Marital status*	
Single	27 (75.0%)
Married/Civil partner	2 (5.6%)
Divorced/Person whose civil partnership has dissolved	7 (19.4%)
*Main offence*	
Sexual offences	19 (52.8%)
Violence against person	12 (33.3%)
Other miscellaneous offences	3 (8.3%)
Robbery	1 (2.8%)
Preferred not to say	1 (2.8%)
*Trouble sleeping before coming to prison*	
Yes	24 (66.7%)
No	12 (33.3%)
*Insomnia type*	
Persistent insomnia (>3 months)	35 (97.2%)
Acute insomnia (≤3 months)	1 (2.8%)
On at least one medication for physical or mental health	
Yes	29 (80.6%)
No	7 (19.4%)
Type of medication	
Antipsychotic or antidepressants^b^	10 (34.5%)
Others^b^	19 (65.5%)
*Mood (PHQ-9 cutoff ≥10) (n = 35) N %*	
Above	32 (91.4%)
Below	3 (8.6%)
*Anxiety (GAD-7 cutoff ≥10) N %*	
Above	24 (66.7%)
Below	12 (33.3%)
Insomnia (SCI)^a^	5.8 ± 3.6
Sleep quality (PSQI)^a^	14.1 ± 2.0
Fatigue (FFS)^a^	14.2 ± 8.
Sleepiness (ESS)^a^	5.9 ± 4.1
Mood (PHQ-9) (*n* = 35)^a^	16.2 ± 5.0
Well-being (WEMWBS)^a^	35.1 ± 9.5
Anxiety (GAD-7)^a^	12.2 ± 5.4
Suicide ideation (BSS)^a^	3.4 ± 5.7
General health (PROMIS)^a^	12.8 ± 2.9
Cognitive failures (CFQ)^a^	36.2 ± 12.7

^a^Statistics are mean ± standard deviation.

^b^of *n* = 29 participants who were currently prescribed medication. SCI, sleep condition indicator; PSQI, Pittsburgh Sleep Quality Index; FFS, flinders fatigue scale; ESS, Epworth Sleepiness Scale; PHQ-9, patient health questionnaire; WEMWBS, Warwick-Edinburgh Mental Well-being Scale; GAD-7, generalized anxiety disorder assessment; PROMIS-10, patient-reported outcomes measurement information system 10-question short form; CFQ, cognitive failures questionnaire; BSS, Beck Suicide Scale.

### Overview of those who completed the treatment pathway

At baseline, all participants (36/36; 100%) had clinically significant insomnia symptoms (SCI ≤16) and over half (16/28; 57.0%) reported clinically significant symptoms at post-assessment. Treatment uptake was moderate (19/36; 52.8%). Just under half of those who the completed baseline assessment left the pathway at the self-management with peer support stage (17/36; 47.2%), and an additional two left with environmental aids because their sleep significantly improved. The remaining participants received CBTi (11/36; 30.6%) and all completed the full course, consisting of at least six CBTi sessions. Sleep hygiene and strategies to minimize nighttime disruption were omitted for three participants because they did not form part of the maintenance formulation for their insomnia. The mean treatment duration was 28 days for self-management with peer support, 35 days for those who additionally used environmental aids, and 121 days for those who continued to CBTi. Post-assessments were completed at the earliest opportunity. Most participants completed assessments before and after the pathway indicating a possible high retention (28/36; 77.8%). There were no SAEs and no ACCTs were opened during the study. All participants who were interviewed (n=10) recommended the treatment pathway.

### Insomnia symptoms across whole pathway

Almost all participants who completed both pre and post-assessments improved their sleep after having the intervention (27/28, 96.4%). The pre to post-pathway change in SCI score was in the large effect size range (*d* = 1.81; 95% confidence intervals, CI: 8.3 to 12.9) (Table 4). The PSQI data supported the finding of a large effect size improvement in sleep from baseline to outcome assessment (*d = *−1.01; CI: −2.6 to −6.0) (Table 4). Seven participants produced complete actigraphy data (7/10; 70%). Of these, effect sizes were low across all three parameters (Table 4). Overall, there was a disagreement between actigraphy data and subjective data (PSQI and SCI) (Supplementary file). One participant who left the pathway after CBTi had agreement between subjective and actigraphy data.

**Table 4.** Subjective (*n* = 28) and Objective Sleep Parameters (*n* = 7) Before and After the Pathway

Sleep parameter	Baseline	Post-intervention	Mean difference (95% CI)	Effect size (*d*)
*Objective actigraphy sleep parameters*				
Total sleep time (min)	488.7 (204.8)	424.8 (167.3)	−20.4 (−43.5 to −84.3)	−0.09
Time in bed (min)	814.5 (332.4)	706.0 (302.0)	−49.6 (−52.2 to −151.4)	−0.14
Sleep efficiency (%)	64%	66%	1.38 % (10.2 to 7.5)	0.05
*Subjective self-report*				
SCI^a^^,^^b^	5.5 ± 3.5	16.1 ± 5.9	10.6 (8.3 to 12.9)	1.81
PSQI^a^	14.0 ± 2.2	9.7 ± 4.0	−4.3 (−2.6 to −6.0)	−1.01
FFS^a^	14.9 ± 8.0	9.8 ± 6.3	−5.1 (−2.5 to −7.7)	−0.77
ESS^a^	6.5 ± 4.1	5.0 ± 3.2	−1.5 (−0.2 to −2.8)	−0.45

^a^Statistics are mean ± standard deviation.

^b^Higher scores indicate better sleep. CI, confidence intervals; SCI, Sleep Condition Indicator; PSQI, Pittsburgh Sleep Quality Index; FFS, Flinders Fatigue Scale; ESS, Epworth Sleepiness Scale.

### Insomnia symptoms by pathway component

SCI mean score improved in those (*n* = 15) who exited the pathway at the self-management with peer-support stage (pretreatment *M* = 5.5 ± 3.7 vs. posttreatment *M* = 16.0 ± 5.7) (Supplementary Material). Just over half reported clinically significant symptoms at post-assessment (8/15, 53.3%) and effect sizes for SCI and PSQI were large (*d* = 1.61; CI: 6.9 to 14.2 and *d* = −0.87; CI: −1.4 to −7.1, respectively). Two-thirds of those receiving CBTi also had clinically significant insomnia symptoms after CBTi (7/11, 63.6%). CBTi demonstrated a large effect size improvement in SCI insomnia (*d* = 1.91; CI: 7.0 to 14.6), which was similar to the overall pathway (*d* = 1.81).

### Health-related outcomes as a whole and by pathway component

Post-assessments showed a reduction in positive screens for both depression (32/35, 91.4% to 12/28, 42.9%) and anxiety (24/36, 66.7% to 8/27 29.6%). PHQ-9, WEMWBS, GAD-7, BSS, PROMIS, and CFQ mean scores all improved from pre- to post-assessment (Table 5). The results indicate a large treatment pathway benefit on depression, anxiety, and cognitive failures, with a medium benefit on suicide ideation (Table 5). This pattern was similar across the self-management with peer support and CBTi groups. However, overall, effect sizes were higher in the CBTi group, and PHQ-9, WEMWBS, GAD-7, and CFQ effect sizes exceeded that which is deemed large (*d = *.80) (Supplementary Material).

**Table 5.** Comparison of Mood, Well-being, and Other Health-Related Outcomes Before and After the Pathway (*n* = 28^*^)

	Baseline mean (SD)	Post-intervention mean (SD)	Mean difference (95% CI)	Effect size (*d*)
PHQ-9	16.6 ± 4.8	9.0 ± 4.3	−7.6 (−5.4 to −9.8)	−1.33
WEMWBS^a^	34.6 ± 9.8	44.1 ± 8.6	9.5 (5.9 to 13.1)	1.02
GAD-7 (*n* = 27)	12.1 ± 5.5	7.3 ± 4.9	−4.9 (−2.8 to −6.9)	−0.94
BSS	3.6 ± 6.1	1.6 ± 3.4	−1.9 (−0.3 to −3.5)	−0.47
PROMIS^a^	12.4 ± 2.7	13.9 ± 2.6	1.5 (0.6 to 2.4)	0.65
CFQ (*n* = 27)	38.8 ± 11.5	28.3 ± 12.0	−10.5 (−5.6 to −15.4)	-0.85

^a^Higher scores indicate an improvement.

^*^Sample size unless otherwise stated. PHQ-9, Patient Health Questionnaire; WEMWBS, Warwick-Edinburgh Mental Well-being Scale; GAD-7, Generalized Anxiety Disorder Assessment; PROMIS-10, Patient-reported outcomes measurement information system 10-question short form; CFQ, Cognitive Failures Questionnaire; BSS, Beck Suicide Scale.

## Discussion

This is the first study to assess the feasibility of a treatment pathway for chronic insomnia in prison to inform a full RCT. Despite the challenging prison environment, we met our feasibility criteria on eligibility, uptake of CBTi, and assessment completions; it was feasible to deliver a non-pharmacological stepped-care treatment pathway and test it using a pre-post study design. People who were incarcerated were able to give informed consent, there was a high recruitment rate and everyone who was offered CBTi completed the full course of therapy. Retaining people who were incarcerated in earlier parts of the treatment pathway was more difficult because of practical (i.e. transferred out of prison) or health-related issues (i.e. transferred to healthcare team to look after other health conditions). Nevertheless, most of those who left the pathway completed follow-up assessments, which suggests there is a desire to participate. Participants engaged with the self-management sleep booklets and stimulus control adaptations (e.g. not lying on the bed during the day). Overall, while the treatment pathway was deemed feasible, session delivery at the prespecified time points was challenging and took longer than anticipated across all stages (e.g. CBTi). This was due to changes in the prison schedule, patient unavailability, and prison lockdowns; all meaning the therapist could not see the patient.

Initial pre-post efficacy data indicates that the stepped-care treatment pathway led to improvements in sleep demonstrated by large effect sizes. The treatment benefits and encouraging recovery rates for depression and anxiety after receiving insomnia treatment suggest the approach could be of further benefit to a population who have mental health conditions [47], though this requires testing in a controlled trial. In addition, there is current concern about the elevated deaths by suicide in the prison population, and in comparison, to the general population [48, 49]. The reduction in suicidal ideation following insomnia treatment in this study, is therefore promising; however, caution should be applied when interpreting this result because the wide confidence intervals span a range from the treatment increasing or decreasing suicidal ideation. A larger controlled trial is required to estimate the insomnia treatment effect on suicidal ideation with greater precision. These findings fit with the broader literature indicating that sleep disruption is a contributory cause of mental health problems [5, 50], particularly affective symptoms (e.g. depression). As such, sleep is now being targeted using non-pharmacological treatment (e.g. CBTi) in community psychiatric services and has seen similar positive effects on depression recovery [51].

Self-management with peer support (the lower intensity intervention) benefits from lower resource use and therefore cost. There was an initial indication that the more intensive treatment option (CBTi) may lead to slightly larger effect sizes. However, the CBTi group also had a larger treatment window and larger dose of treatment. An adequately powered dismantling study with a health economic evaluation is needed to test the individual treatment effects. This type of study could optimize the pathway design and potentially it’s efficiency.

The lack of overall change in actigraphy-measured total sleep time and sleep efficiency was largely discrepant with the subjective sleep outcomes across all pathway stages. However, most studies that examine CBTi treatment effects using objective measures find that there is no change in sleep parameters over time [52]. It is also possible that the sedentary nature of prison life might decrease the accuracy of the actigraphy watch to detect distinct sleep–wake periods, as has been reported in other trials [53]. In addition, the amount of missing sleep diary data meant we had to rely on comparison with PSQI and SCI data only. Going forward, a larger study could investigate factors associated with improvement in the objective and subjective sleep outcomes.

### Strengths and limitations

To the best of our knowledge, our study is the first to examine a stepped-care treatment pathway for insomnia, and specifically CBTi, in people who are incarcerated with chronic insomnia. Subjective and objective measures were used to examine sleep in prison for the first time. Importantly, the experiences and voices of people who have been, or are currently incarcerated were central to the study, from the design of the pathway, and co-production of self-management sleep booklets through to delivery of the intervention with peer support workers; this likely helped to facilitate treatment engagement. However, the study also had limitations. Firstly, we did not have a control group. Therefore, we were unable to control for the effects of other variables (e.g. medication treatment), and large effect sizes are likely to be inflated. Secondly, we had no follow-up assessment, therefore, we do not know longer-term effects. Thirdly, the varied time periods for assessments mean it is uncertain which period gives the most benefit to the sleep of people who were incarcerated. Fourthly, the data was self-reported and therefore may be potentially subject to self-report bias. People who are incarcerated may also have been vulnerable to social desirability bias in line with previous studies. Both potential biases may have impacted the internal validity of the study. Lastly, as only one high-secure male prison setting was included our findings might not be generalizable. Future research should look at different categories of prison establishments (e.g. female prisons, young offenders institutions) and lower severity offence types (e.g. theft, drug related) as they may respond to the treatment differently or may need further adaptation. An example may be to adapt the self-management sleep booklets for people who cannot read, or in other languages to improve the potential for global impact.

The non-pharmacological sleep treatment approach represents a radical shift from current insomnia treatment offered in prisons. It overcomes risks of medication trading, misuse and selling, and barriers to its on-wing medicine administration. The treatment approach was popular with people who were incarcerated and was associated with large within-participants improvements in sleep and mental health but requires further testing in a controlled trial. Common treatment barriers were logged by the therapist (e.g. high-level co-morbid physical health problems, substance misuse, and medication effects) and the treatment could be refined considering these. Notably, unprecedented changes to prison routine (e.g. prison lockdown) in the real-world setting means the therapist must be flexible in their treatment approach.

Two-thirds of people who are incarcerated experience insomnia. A non-pharmacological sleep intervention is feasible and may be efficacious for targeting chronic insomnia in this setting. However, flexibility is needed in approaching session delivery due to prison schedule uncertainty. Treating sleep may be an additional non-stigmatizing route to improving the mental health and reducing suicidality of people who are incarcerated. A larger controlled pragmatic trial of the intervention with economic modeling is the clear next step.

## Supplementary Material

zpae003_suppl_Supplementary_Material

## Footnotes

1 Escape would be highly dangerous to the public or the police or the security of the State and for whom the aim must be to make escape impossible’ (Ministry of Justice, 2011 pp 6).

2 HM Prison Service care planning process specifically for those at risk of suicide or self-harm.

3 1 missing data.
