# Supplementary material for: Treating insomnia in people who are incarcerated: a feasibility study of a multicomponent treatment pathway
Source: Sleep Adv. 2024 Jan 20;5(1):zpae003. doi: 10.1093/sleepadvances/zpae003 (PMC10873789; doi:10.1093/sleepadvances/zpae003)
Supplement: zpae003_suppl_Supplementary_Material [file zpae003_suppl_supplementary_material.docx]

Treating insomnia in people who are incarcerated: a feasibility study of a multi-component treatment pathway

Lindsay H Dewa^1^

Bethan Thibaut^1^

Natalie Pattison^2^

Sean James Campbell*^3^

Thomas Woodcock^1^

Paul Aylin^1^

Stephanie Archer^4^

^1^ Imperial College London, School of Public Health, London, UK

^2^ Care UK, London, UK

^3^ User Voice, London, UK

^4^ University of Cambridge, UK

Corresponding author: Dr Lindsay H Dewa, Imperial College London, School of Public Health, Charing Cross Hospital, Reynolds Building, St Dunstan’s Road, London, W6 8RP [l.dewa@imperial.ac.uk](mailto:l.dewa@imperial.ac.uk)

*On behalf of User Voice

Supplementary files

Table S1: Self-management with peer support pathway components means (SD) and effect sizes baseline and post assessment (n=15)

| Variable | Baseline | Post assessment | Mean difference  (95% CI) | d=M/SD | Effect size |
| --- | --- | --- | --- | --- | --- |
| SCI | 5.5 ± 3.7 | 16.0 ± 5.7 | 10.5  (6.9 to 14.2) | 1.61 | Large |
| PSQI | 14.2 ± 1.9 | 9.9 ± 4.3 | -4.3  (-1.4 to -7.1) | -0.87 | Large |
| FFS | 14.3 ± 9.0 | 9.5 ± 7.8 | -4.8  (-1.5 to -8.1) | -0.80 | Large |
| ESS | 4.8 ± 3.3 | 3.9 ± 3.0 | -0.9  (-3.0 to 1.1) | 0.26 | Small |
| PHQ-9 | 16.4 ± 4.9 | 9.1 ± 4.6 | -7.3  (-4.2 to -10.3) | -1.31 | Large |
| GAD-7 | 11.1 ± 5.8 | 5.8 ± 5.4 | -5.3  (-2.1 to -8.4) | -0.97 | Large |
| WEMWBS | 33.6 ± 8.0 | 43.5 ± 8.9 | 9.9  (4.9 to 15.0) | 1.09 | Large |
| PROMIS | 12.4 ± 3.1 | 13.5 ± 3.1 | 1.1  (-0.2 to 2.5) | -0.47 | Small |
| CFQ | 36.9 ± 12.3 | 29.9 ± 13.1 | -7.0  (-14.7 to 0.7) | 0.53 | Moderate |
| BSS | 1.9 ± 3.6 | 0.5 ± 1.4 | -1.4  (-3.0 to 0.2) | -0.50 | Moderate |

SCI Sleep Condition Indicator; PSQI Pittsburgh Sleep Quality Index; FFS Flinders Fatigue Scale; ESS Epworth Sleepiness Scale; PHQ-9, Patient Health Questionnaire; WEMWBS Warwick-Edinburgh Mental Wellbeing Scale; GAD-7 Generalised Anxiety Disorder Assessment; PROMIS-10 Patient-reported outcomes measurement information system 10-question short form; CFQ Cognitive Failures Questionnaire; BSS Beck Suicide Scale.

Table S2: CBTi pathway components means (SD) and effect sizes baseline and post assessment (n=11)

| Variable | Baseline | Post assessment | Mean difference | d=M/SD | Effect size |
| --- | --- | --- | --- | --- | --- |
| SCI | 5.3 ± 3.5 | 16.1 ± 7.0 | 10.8  (7.0 to 14.6) | 1.91 | Exceeds large |
| PSQI | 14.2 ± 2.2 | 9.4 ± 4.1 | -4.8  (-2.2 to -7.4) | -1.25 | Exceeds large |
| FFS | 14.9 ± 7.6 | 9.6 ± 4.5 | -5.4  (-0.1 to -10.8) | -0.66 | Moderate |
| ESS | 8.5 ± 4.6 | 6.9 ± 2.9 | -1.6  (0.2 to -3.3) | -0.58 | Moderate |
| PHQ-9 | 17.6 ± 4.8 | 9.2 ± 4.3 | -8.5  (-4.1 to -12.8) | -1.32 | Exceeds large |
| GAD-7 | 13.9 ± 5.1 | 9.1 ± 4.2 | -4.8  (-1.2 to -8.4) | -0.90 | Large |
| WEMWBS | 32.0 ± 8.4 | 43.3 ± 8.4 | 11.3  (5.2 to 17.3) | 1.25 | Exceeds large |
| PROMIS | 12.5 ± 2.5 | 14.5 ± 2.2 | 2.1  (0.4 to 3.7) | 0.85 | Large |
| CFQ | 44.0 ± 8.0 | 28.5 ± 10.8 | -15.5  (-8.1 to -22.8) | -1.41 | Exceeds large |
| BSS | 6.5 ± 8.1 | 3.5 ± 4.7 | -3.0  (0.8 to -6.8) | -0.53 | Moderate |

SCI Sleep Condition Indicator; PSQI Pittsburgh Sleep Quality Index; FFS Flinders Fatigue Scale; ESS Epworth Sleepiness Scale; PHQ-9, Patient Health Questionnaire; WEMWBS Warwick-Edinburgh Mental Wellbeing Scale; GAD-7 Generalised Anxiety Disorder Assessment; PROMIS-10 Patient-reported outcomes measurement information system 10-question short form; CFQ Cognitive Failures Questionnaire; BSS Beck Suicide Scale.

Table 3: Participant level objective actigraphy sleep parameters with subjective SCI and PSQI scores

|  |  | Participant^[[1]](#footnote-1)^ | | | | | | |
| --- | --- | --- | --- | --- | --- | --- | --- | --- |
|  |  | Mark | Mohammed | David | Anthony | Paul | Michael | John |
|  | Pathway exit stage | CBTi | Self-management with peer support | CBTi | Self-management with peer support | Self-management with peer support | Environmental aids | CBTi |
| Total Sleep Time (minutes) | Pre | 429 | 611 | 448 | 327 | 395 | 288 | 610 |
|  | Post | 573 | 470 | 313 | 419 | 552 | 262 | 426 |
|  | Change | 145 | -141 | -135 | 92 | 156 | -26 | -184 |
| Sleep efficiency % | Pre | 63% | 55% | 95% | 64% | 45% | 70% | 58% |
|  | Post | 76% | 56% | 57% | 86% | 62% | 68% | 62% |
|  | Change | 13% | 1% | -38% | 21% | 17% | -2% | 4% |
| Time in bed (minutes) | Pre | 728 | 1140 | 474 | 490 | 911 | 499 | 1050 |
|  | Post | 843 | 881 | 576 | 489 | 875 | 568 | 750 |
|  | Change | 115 | -257 | 103 | -0.714 | -36.9 | 69.1 | -304 |
| PSQI Total score^[[2]](#footnote-2)^ | Pre | 16 | NA | 10 | 15 | NA | 14 | 14 |
|  | Post | 5 | NA | 8 | 8 | NA | 12 | 15 |
|  | Change | -11 | NA | -2 | -7 | NA | -2 | 1 |
| SCI Total score^[[3]](#footnote-3)^ | Pre | 3 | 9 | 8 | 7 | NA | 6 | 7 |
|  | Post | 25 | 16 | 15 | 21 | NA | 16 | 16 |
|  | Change | 22 | 7 | 7 | 14 | NA | 10 | 9 |

SCI Sleep Condition Indicator; PSQI Pittsburgh Sleep Quality Index

1. Participant name made up [↑](#footnote-ref-1)
2. Lower score indicates better sleep [↑](#footnote-ref-2)
3. Higher score indicates better sleep [↑](#footnote-ref-3)
